# Incidence of *Cercopithifilaria bainae* in Dogs and Probability of Co-Infection with Other Tick-Borne Pathogens

**DOI:** 10.1371/journal.pone.0088198

**Published:** 2014-02-03

**Authors:** Rafael Antonio Nascimento Ramos, Alessio Giannelli, Riccardo Paolo Lia, Emanuele Brianti, Viviana Domenica Tarallo, Edward B. Breitshwerdt, Filipe Dantas-Torres, Dorothee Stanneck, Domenico Otranto

**Affiliations:** 1 Dipartimento di Medicina Veterinaria, Università degli Studi di Bari, Valenzano, Italy; 2 Dipartimento di Sanità Pubblica Veterinaria, Facoltà di Medicina Veterinaria, Università degli Studi di Messina, Messina, Italy; 3 Intracellular Pathogens Research Laboratory, College of Veterinary Medicine, North Carolina State University, Raleigh, North Carolina, United States of America; 4 Departamento de Imunologia, Centro de Pesquisas Aggeu Magalhães (Fiocruz-PE), Recife, Pernambuco, Brazil; 5 Bayer Animal Health GmbH, Leverkusen, Germany; University of Texas Medical Branch, United States of America

## Abstract

**Background:**

*Cercopithifilaria bainae* is a filarioid parasite that infects dogs, being transmitted by *Rhipicephalus sanguineus* group ticks in many countries of the Mediterranean basin. This study assessed the incidence density rate (IDR) of infection by *C. bainae* in dogs and the probability of co-infection with other tick-borne pathogens (i.e., *Anaplasma platys*, *Babesia vogeli* and *Hepatozoon canis*), in an area of high endemicity in southern Italy.

**Methodology/Principal Findings:**

From March 2011 to October 2012, a field study involving 58 young dogs naturally exposed to tick infestation was conducted. Skin and blood samples obtained from each dog six times during an 18-month period were tested for *C. bainae* by parasite detection within skin snip sediments, with subsequent confirmation through PCR and DNA sequencing. Dogs examined monthly for ticks and *A. platys*, *B. vogeli* and *H. canis* were microscopically and/or molecularly diagnosed and after the first and the second summer seasons, the IDR for positive animal-month at risk was 3.8% and 1.7% in November 2011 and October 2012, respectively. All 58 *C. bainae*-infected dogs were simultaneously infected with at least one other tick-borne pathogen. After the first summer season (assessment in November 2011), a *C. bainae*-infected dog had a 33% probability of being infected with *H. canis* or *A. platys*, whereas after the second tick season (assessment in October 2012) the probability of co-infection was 78%, 22% and 11% for *H. canis*, *A. platys* and *B. vogeli*, respectively.

**Conclusions:**

Our data indicate that tick-infested dogs are at risk of acquiring infection by *C. bainae*. In addition, the detection of *C. bainae* microfilariae indicates a prior tick exposure and, should stimulate testing for other tick-borne disease causing pathogens.

## Introduction

The genus *Cercopithifilaria* comprises 28 species of filarial nematodes that live beneath the skin of wild and domestic mammals (e.g., ruminants, cercopithecid primates, carnivores, rodents and marsupials) [1]. Dogs may be infected by *Cercopithifilaria grassii*, *Cercopithifilaria* sp. II and *Cercopithifilaria bainae* [2], [3], [4], with the latter species having a prevalence of infection reaching as high as 45.4% in some dog populations from the Mediterranean area [2], [5]. Indeed, the distribution of *C. bainae* overlaps that of tick vectors belonging to the *Rhipicephalus sanguineus* group. In the vector, microfilariae reach the infective larval stage (L3) within approximately 30 days [6], [7].

Although the pathological relevance of this filarioid has not been clearly defined, histological findings suggest that microfilariae may cause mild focal epidermal and subepidermal edematous changes and a perivascular interstitial dermatitis, characterized by the presence of neutrophils, eosinophils and lymphocytes [8]. Moreover, it is not clear whether the infection by *C. bainae* affects the dog's immune response to other tick-borne pathogens or *vice versa*, facilitating their acquisition during the tick bite. However, given the paucity of data available about the biological behavior of this nematode under natural conditions, this study aimed to assess, for the first time, the incidence of *C. bainae* infection in dogs naturally exposed to *R. sanguineus* group ticks and to examine the probability of co-infections with other tick-borne pathogens (i.e., *Anaplasma platys*, *Babesia vogeli* and *Hepatozoon canis*), in dogs residing in an area of high tick infestation pressure from southern Italy.

## Materials and Methods

This study was carried out in a private shelter in the municipality of Putignano (40°51′N, 17°07′E), province of Bari, Apulia region, southern Italy. This area has a high prevalence of canine tick-borne pathogens and dogs maintained in this shelter were constantly exposed to ticks [9], [10].

This research was an extension of studies previously performed [8], [11]. Sampled animals (n = 58) included young dogs (≤6 months old) of both genders, which were dewormed (Drontal plus®; Bayer AG, Germany) and vaccinated (Duramune® DAPPI + LC; Fort Dodge Animal Health, Italy) at their enrolment (March-May 2011). Each dog was clinically examined, searched for ticks, and sampled for skin and blood six times during the study (i.e., at the baseline in March-May 2011, in July, September, November 2011, and April, October 2012), following the procedures previously described [11], [12]. Dogs were examined monthly (from March 2011 to October 2012) for the presence of ticks by thumb counting of the following body regions: head, ears, breast-neck, thorax, abdomen, fore and back limbs, inter-digital areas, armpit, tail and inguinal area [11], and ticks collected were morphologically identified [13], [14].

*Cercopithifilaria* spp. larvae were detected by microscopic examination of skin snips sampled from the inter-scapular region, soaked in saline solution (pH 7.4) and processed as described elsewhere [8]. Briefly, skin snips were soaked in saline solution for about 6 hours at room temperature, and the sediment (20 µl) was examined microscopically at different magnifications (10 and 40×). The microfilariae species were identified morphologically and confirmed by PCR amplification and DNA sequencing [3], [8]. Infections with other tick-borne pathogens (i.e., *A. platys*, *B. vogeli* and *H. canis*) were confirmed by cytological examination of stained blood smears or by PCR amplification and DNA sequencing of selected amplicons, as described elsewhere [11].

The incidence density rate (IDR) of infection was calculated based on the number of dogs infected with *Cercopithifilaria* spp. larvae at each sampling time point divided by the number of dog-months of follow-up, as previously described [11]. In addition, co-infections with other pathogens were statistically analyzed at two sampling times following the peak of tick infestation (i.e., November 2011 and October 2012), without considering transient positivity that may have occurred at *interim* sampling times. The conditional probability of co-infections was calculated by assessing the absolute frequency of positive animals, based on the Bayes' theorem using the statistical software R version 2.15.2 [15].

The study was conducted according to the principles of Good Clinical Practice (VICH GL9 GCP, 2000 http://www.emea.eu.int/pdfs/vet/vich/059598en.pdf) in the guideline for the testing and evaluation of the efficacy of antiparasitic substances for the treatment and prevention of tick and flea infestation in dogs and cats (http://www.emea.eu.int/pdfs/vet/ewp/000500en.pdfl) and the guideline on Statistical Principles for Veterinary Clinical Trials (CVMP/816/00, 2000 www.emea.eu.int/pdfs/vet/ewp/081600en.pdf). The study design and the experimental procedures were approved and authorized by the Italian Ministry of Health (authorization number DGSA n°. 0001997; 04/02/2011).

## Results and Discussion

**Figure 1:**
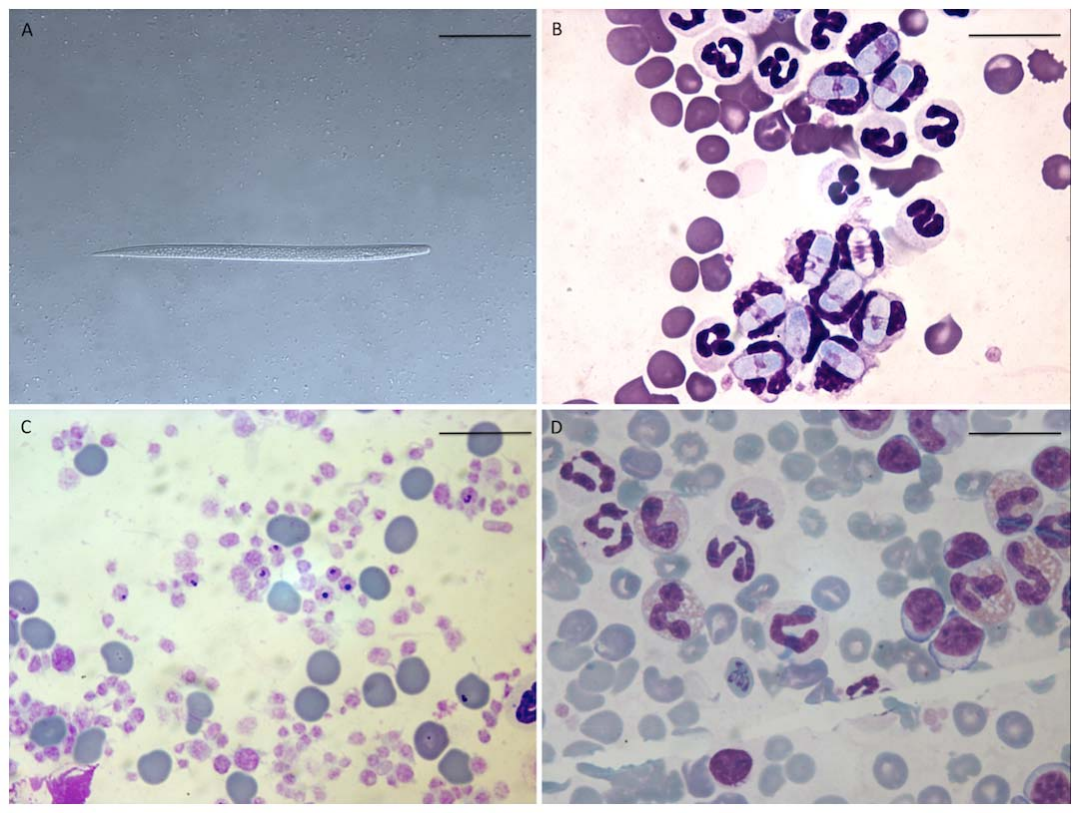
Tick-borne pathogens identified during the study. (A) First-stage larvae (L1) of *Cercopithifilaria bainae* retrieved in a skin snip of a dog (scale-bar = 50 µm); (B) *Hepatozoon canis* gamont (scale-bar = 20 µm); (C) *Anaplasma platys* (scale-bar = 20 µm).; (D) *Babesia vogeli* trophozoites retrieved at blood-smear examination (scale-bar = 20 µm).

At baseline (March-May 2011), *Cercopithifilaria* spp. microfilariae were not visualized in skin snips from any of the young dogs enrolled. The highest IDR values for positive animal-month at risk (Table 1), were found in November 2011 and October 2012, (i.e., 3.8% and 1.7%, respectively), after the first and the second summer seasons, which, in turn, correspond to the highest number of ticks found on dogs in the study area [9]. Based on morphological characteristics, PCR amplification and sequencing (data not shown), microfilariae were identified as *C. bainae* (Fig. 1A). New cases of *C. bainae* infection in dogs occurred throughout the year in the study area, in accordance with the ongoing presence of the tick vector. The finding of dermal microfilariae at the first sampling time (July 2011) in animals younger than 6 months of age indicates that the pre-patent period of *C. bainae* is short, at least less than 6 months, as suggested in a previous study [8]. In addition, the highest IDR found in November 2011 (i.e., 3.8%) occurred six months after the time (i.e., June), in which the highest prevalence of ticks harboring infective stages of *C. bainae* was confirmed by tick dissection [16]. Although requiring confirmation, this period of prepatency is similar to that reported for *Cercopithifilaria rugosicauda*, which infects roe deer in Europe [17].

**Table 1:** Incidence density rate (IDR) of positive animal-month at risk for *Cercopithifilaria bainae*-infected dogs throughout the study period.

Sampling time	Number of dogs*	Number of new cases	Dog-months of follow-up	IDR
Baseline (March-May 2011)	58	0	–	–
July 2011	58	2	174	1.1
September 2011	53	0	106	0
November 2011	52	4	104	3.8
April 2012	51	4	255	1.5
October 2012	48	5	288	1.7

* These numbers represent the negative dogs that have been included in the cohort for calculating the IDR at each sampling time.

All 15 *C. bainae*-infected dogs were also cytologically and/or PCR-positive for other tick-borne pathogens (i.e., *A. platys*, *B. vogeli* and *H. canis*) at one or more follow-up time points. Of four dogs infected with *C. bainae* in November 2011, two were infected with *H. canis* (Fig. 1B) and the other two with *A. platys* (Fig. 1C). Therefore, based on the Bayes' theorem, a dog positive for *C. bainae* presented a 33% probability of being infected with *H. canis* or *A. platys*. After the second tick season (October 2012), co-infections were documented in nine dogs, four of which were new *C. bainae* cases. Seven of nine dogs were co-infected with *H. canis* (77.7%), and two dogs (22.2%) were co-infected with *A. platys*, of which one was also infected with *B. vogeli* (11.1%; 1/9) (Fig. 1D). Therefore, the probability that a *C. bainae*-infected dog was concurrently infected with another tick-borne pathogen was 78%, 22% and 11% for *H. canis*, *A. platys* and *B. vogeli*, respectively. Of 48 and 39 animals negative for *C. bainae* in November 2011 and October 2012, respectively, 17 (35.4 %) and 21 (53.8 %) were positive for one or more TBD causing pathogen.

Co-infections in dogs with pathogens transmitted by ticks is a frequent occurrence in southern Italy [11], [18] and this study indicates that *C. bainae*-infected dogs are at risk for prior, current or future co-infections with other pathogens (i.e., *A. platys*, *B. vogeli* and *H. canis*). The high probability that *C. bainae*-infected dogs were co-infected with *H. canis* during the first (33%) and second (78%) tick seasons also indicates a high risk of environmental tick infestation. Although all dogs were infested by *R. sanguineus* group ticks during any time within the study period, the highest prevalence of tick infestation in *C. bainae*-positive dogs (100%; 8/8) was recorded in April, May and August 2012, whereas the lowest infestation level (25%; 1/4) was in January and February of the same year, when ticks are less prevalent in this area [9].

In spite of erythematous and papular dermatitis reported in infected animals [8], the pathogenic role of *C. bainae* in dogs remains to be elucidated. Although this filarioid is regarded as minimally pathogenic, the detection of dermal microfilariae provides evidence for a previous tick infestation and should stimulate consideration of infections with other tick-borne pathogens. However, whether *C. bainae* infection adversely affects the dog's immune response to other TBD causing pathogens, or *vice versa*, deserves to be studied.

## References

[1] Bain O, Uni S, Takaoka H (2002) A synthetic look at a twenty years old taxon, *Cercopithifilaria* its probable evolution. In: Proceedings of the 10th International Congress of Parasitology (ICOPA) Vancouver: Monduzzi Editore. pp. 365–368.

[2] Otranto D, Brianti E, Dantas-Torres F, Weigl S, Latrofa MS, et al (2011) Morphological and molecular data on the dermal microfilariae of a species of *Cercopithifilaria* from a dog in Sicily. Vet Parasitol 182: 221–229. 21705146 10.1016/j.vetpar.2011.05.043

[3] Otranto D, Brianti E, Dantas-Torres F, Miró G, Latrofa MS, et al (2012) Species diversity of dermal microfilariae of the genus *Cercopithifilaria* infesting dogs in the Mediterranean region. Parasitology 140: 99–108. 22914299 10.1017/S0031182012001357

[4] Otranto D, Varcasia A, Solinas C, Scala A, Brianti E, et al (2013) Redescription of *Cercopithifilaria bainae* Almeida & Vicente, 1984 (Spirurida, Onchocercidae) from a dog in Sardinia, Italy. Parasit Vectors 6: 132. 23642161 10.1186/1756-3305-6-132 PMC3655055

[5] Otranto D, Brianti E, Latrofa MS, Annoscia G, Weigl S, et al (2012) On a *Cercopithifilaria* sp. transmitted by *Rhipicephalus sanguineus*: a neglected, but widespread filarioid of dogs. Parasit Vectors 5: 1. 22212459 10.1186/1756-3305-5-1 PMC3259067

[6] Brianti E, Otranto D, Dantas-Torres F, Weigl S, Latrofa MS, et al (2012) *Rhipicephalus sanguineus* (Ixodida, Ixodidae) as intermediate host of a canine neglected filarial species with dermal microfilariae. Vet Parasitol 10: 330–337. 10.1016/j.vetpar.2011.07.031 21831524

[7] Ramos RA, Giannelli A, Brianti E, Annoscia G, Cantacessi C, et al (2013) Tick vectors of *Cercopithifilaria bainae* in dogs: *Rhipicephalus sanguineus* sensu lato versus *Ixodes ricinus* . Parasitol Res 112: 3013–3017. 23771741 10.1007/s00436-013-3474-4

[8] Otranto D, Brianti E, Abramo F, Gaglio G, Napoli E, et al (2012) Cutaneous distribution and localization of *Cercopithifilaria* sp. microfilariae in dogs. Vet Parasitol 190: 143–150. 22698796 10.1016/j.vetpar.2012.05.016

[9] Lorusso V, Dantas-Torres F, Lia RP, Tarallo VD, Mencke N, et al (2010) Seasonal dynamics of the brown dog tick, *Rhipicephalus sanguineus*, on a confined dog population in Italy. Med Vet Entomol 24: 309–315. 20557458 10.1111/j.1365-2915.2010.00885.x

[10] Dantas-Torres F, Otranto D (2011) *Rhipicephalus sanguineus* on dogs: relationships between attachment sites and tick developmental stages. Exp Appl Acarol 53: 389–389. 20957414 10.1007/s10493-010-9406-4

[11] Dantas-Torres F, Capelli G, Giannelli A, Ramos RA, Lia RP, et al (2013) Efficacy of an imidacloprid/flumethrin collar against fleas, ticks and tick-borne pathogens in dogs. Parasit Vectors 6: 245. 23972013 10.1186/1756-3305-6-245 PMC3766024

[12] Otranto D, Dantas-Torres F, de Caprariis D, Di Paola G, Tarallo VD, et al (2013) Prevention of canine leishmaniosis in a hyper-endemic area using a combination of 10% imidacloprid/4.5% flumethrin. PLoS One 8: e56374. 23451043 10.1371/journal.pone.0056374 PMC3581506

[13] Walker JB, Keirans JE, Horak IG (2000) The genus *Rhipicephalus* (Acari, Ixodidae): a guide to the brown ticks of the world. Cambridge: Cambridge University Press.

[14] Dantas-Torres F, Latrofa MS, Annoscia G, Giannelli A, Parisi A, et al (2013) Morphological and genetic diversity of *Rhipicephalus sanguineus* sensu lato from the New and Old Worlds. Parasit Vectors 6: 213. 23880226 10.1186/1756-3305-6-213 PMC3735430

[15] R Core Team (2012) R: A language and environment for statistical computing. Vienna: R Foundation for Statistical Computing.

[16] Ramos RA, Giannelli A, Carbone D, Baneth G, Dantas-Torres F, et al. (2013) Seasonal occurrence of *Hepatozoon canis* and *Cercopithifilaria bainae* in *Rhipicephalus sanguineus* group ticks from the environment. Ticks Tick-borne Dis “In press”. 10.1016/j.ttbdis.2013.12.005 24594107

[17] Winkhardt HJ (1980) The larval development of *Dipetalonema rugosicauda* (syn, *Wehrdikmansia rugosicauda*) in the tick *Ixodes ricinus*. II. The development of *Dipetalonema rugosicauda* in *Ixodes ricinus* and investigations about the occurrence of the microfilariae in the roe deer (*C. capreolus*). Z Tropenmed Parasitol 31: 21–30. 7189613

[18] De Tommasi AS, Otranto D, Dantas-Torres F, Capelli G, Breitschwerdt EB, et al (2013) Are vector-borne pathogen co-infections complicating the clinical presentation in dogs? Parasit Vectors 6: 97. 23587324 10.1186/1756-3305-6-97 PMC3637325

